# Insights into structure and activity of a UDP-GlcNAc 2-epimerase involved in secondary cell wall polymer biosynthesis in *Paenibacillus alvei*

**DOI:** 10.3389/fmolb.2024.1470989

**Published:** 2024-09-26

**Authors:** Cordula Stefanović, Max S. G. Legg, Nick Mateyko, Jakob J. Ender, Tea Kuvek, Chris Oostenbrink, Christina Schäffer, Stephen V. Evans, Fiona F. Hager-Mair

**Affiliations:** ^1^ Department of Chemistry, Institute of Biochemistry, NanoGlycobiology Research Group, Universität für Bodenkultur Wien, Vienna, Austria; ^2^ Department of Biochemistry & Microbiology, University of Victoria, Victoria, BC, Canada; ^3^ Department of Material Sciences and Process Engineering, Institute of Molecular Modeling and Simulation, Universität für Bodenkultur Wien, Vienna, Austria

**Keywords:** glycobiology, kinetic constants, non-hydrolyzing epimerase, *Paenibacillus alvei*, S-layer anchoring, structure-activity relationship, tunicamycin, X-ray structure

## Abstract

**Introduction:**

S-layer anchoring in *Paenibacillus alvei* is enabled by a non-covalent interaction between an S-layer homology domain trimer and a secondary cell wall polymer (SCWP), ensuring the structural integrity of the bacterial cell wall. Within the SCWP repeat, pyruvylated ManNAc serves as the ligand and the UDP-GlcNAc-2-epimerase MnaA supplies UDP-ManNAc to SCWP biosynthesis.

**Methods:**

To better understand SCWP biosynthesis and identify strategies for inhibiting pathogens with comparable cell wall architecture, like *Bacillus anthracis*, MnaA and rational variants were produced in *E. coli* and their kinetic constants determined. The effect of UDP-GlcNAc as a predicted allosteric activator and tunicamycin as a potential inhibitor of MnaA was tested *in vitro* supported by molecular docking experiments. Additionally, wild-type MnaA was crystallized.

**Results:**

We present the crystal structure of unliganded *P. alvei* MnaA resolved at 2.20 Å. It adopts a GT-B fold consistent with other bacterial non-hydrolyzing UDP-GlcNAc 2-epimerases. A comparison of amino acid sequences reveals conservation of putative and known catalytic and allosteric-site residues in MnaA, which was confirmed through analysis of Q42A, Q69A, E135A and H241A MnaA variants. The kinetic parameters *K*_M_ and *k*_cat_ of MnaA were determined to be 3.91 mM and 33.44 s^-1^ for the forward, and 2.41 mM and 6.02 s^-1^ for the reverse reaction. While allosteric regulation by UDP-GlcNAc has been proposed as a mechanism for enzyme activation, UDP-GlcNAc was not found to be essential for UDP-ManNAc epimerization by *P. alvei* MnaA. However, the reaction rate doubled upon addition of 5% UDP-GlcNAc. Unexpectedly, the UDP-GlcNAc analog tunicamycin did not inhibit MnaA. Molecular docking experiments comparing tunicamycin binding of *P. alvei* MnaA and *Staphylococcus aureus* MnaA, which is inhibited by tunicamycin, revealed different residues exposed to the antibiotic excluding, those at the predicted allosteric site of *P. alvei* MnaA, corroborating tunicamycin resistance.

**Conclusion:**

The unliganded crystal structure of *P. alvei* MnaA reveals an open conformation characterized by an accessible cleft between the N- and C-terminal domains. Despite the conservation of residues involved in binding the allosteric activator UDP-GlcNAc, the enzyme is not strictly regulated by the substrate. Unlike *S. aureus* MnaA, the activity of *P. alvei* MnaA remains unaffected by tunicamycin.

## 1 Introduction

Non-hydrolyzing UDP-GlcNAc 2-epimerases catalyzing the reversible conversion of UDP-*N*-acetylglucosamine (UDP-GlcNAc) and UDP-*N*-acetylmannosamine (UDP-ManNAc) (Velloso et al., 2008; Hager et al., 2018) are central to the biosynthesis of various bacterial cell wall polysaccharides. These include non-classical secondary cell wall polymers (SCWPs) in *e.g*., *Bacillus anthracis* or *Paenibacillus alvei* (Hager et al., 2018; Kern et al., 2010), wall teichoic acids (WTAs) in *e.g*., *Staphylococcus aureus* (Mann et al., 2016) or capsular polysaccharides (CPSs) in *e.g*., *Neisseria meningitidis* serogroup A (Hurlburt et al., 2020), among others. Given the importance of these compounds for the integrity and functionality of the bacterial cell wall, one functional UDP-GlcNAc 2-epimerase ortholog is usually required for vegetative bacterial growth, as shown for instance for *Bacillus anthracis* (Wang et al., 2014) and *S. aureus* where enzyme deletion restored β-lactam antibiotic sensitivity in MRSA (Mann et al., 2016). This observation together with the presence of hydrolyzing bifunctional UDP-GlcNAc 2-epimerase/ManNAc kinases in mammals, in contrast to bacteria (Wang et al., 2014), highlights the bacterial enzyme as a valuable target for the development of novel antimicrobials. Notably, the mammalian enzyme catalyzes the rate-limiting step in sialic acid biosynthesis and is a crucial regulator of cell-surface sialylation in humans (Keppler et al., 1999).

4,6-pyruvateketal-modified *N*-acetylmannosamine (pyrManNAc), a constituent of peptidoglycan-bound SCWPs in various Gram-positive bacteria, serves as a cell wall ligand for cell surface proteins that contain a terminal cell surface (S-) layer homology (SLH) domain trimer. This structural epitope plays a critical role for maintaining the structural integrity of the bacterial cell wall (Schäffer and Messner, 2005). The interaction is prominently exemplified by S-layer proteins, which form 2D crystalline arrays covering many prokaryotic cells (Sleytr et al., 2014; Fagan and Fairweather, 2014). S-layers are virulence factors in several pathogens, including *e.g., B. anthracis*, the etiological agent of anthrax (Missiakas and Schneewind, 2017), *Tannerella forsythia*, a periodontal pathogen (Sakakibara et al., 2007; Sekot et al., 2011), and *Clostridium difficile*, the leading cause of nosocomial infections worldwide (Kirk et al., 2017). The Gram-positive bacterium *P. alvei* CCM 2051^T^, a model organism for studying S-layer anchoring mediated by pyrManNAc, as seen for instance also in the cell wall architecture of *B. anthracis* (Kern et al., 2010; Mesnage et al., 1999; Sychantha et al., 2018; Chapman et al., 2018), anchors its S-layer proteins through a specific, non-covalent interaction between an N-terminal SLH domain trimer (Janesch et al., 2013) and pyrManNAc within its SCWP (Blackler et al., 2018; Legg et al., 2022). The lipid-linked SCWP disaccharide repeat, →3)-4,6-Pyr-β-D-Man*p*NAc-(1→4)-β-D-Glc*p*NAc-(1→3)-(1→ (Schäffer et al., 2000), which constitutes SCWP in *P. alvei*, has previously been synthesized chemo-enzymatically *in vitro* using recombinant *P. alvei* MnaA (PAV_RS07420) to provide UDP-ManNAc for ManNAc incorporation in the SCWP repeat, together with the bacterium’s *N*-acetylmannosamine transferase TagA (PAV_RS07420) and CsaB, which pyruvylates ManNAc within the repeat (PAV_RS07425) (Hager et al., 2018).

The *P. alvei* MnaA enzyme (henceforth abbreviated as *Pa*MnaA) belongs to the UDP-GlcNAc 2-epimerase protein family PF02350 (El-Gebali et al., 2019); structural data from 12 bacterial species are available in the Protein Data Bank (PDB) (Berman et al., 2000) (Table 1). Both, monofunctional bacterial and bifunctional mammalian enzymes (*vide supra*) have similar folds, consisting of two Rossmann-like domains that form an active site in the cleft between them; this structure is characteristic of GT-B fold-type glycosyltransferases (GTs) (Chen et al., 2016). Chemical evidence suggests that the catalytic mechanism of non-hydrolyzing UDP-GlcNAc 2-epimerases involves a two-step elimination-addition mechanism, with a 2-acetamidoglucal intermediate (Tanner, 2002) (Supplementary Figure S1).

**TABLE 1:** Summary of bacterial non-hydrolyzing UDP-GlcNAc 2-epimerase structures in the PDB [modified after (Legg, 2022)].

PDB	Identity to MnaA (%)	Resolution	Organism	Conformation	Catalytic site substrate	Allosteric site substrate	References
3BEO	67.2	1.70 Å	*Bacillus anthracis*	Closed	UDP*	UDP-GlcNAc	Velloso et al. (2008)
1O6C	63.0	2.90 Å	*Bacillus subtilis*	Open	—	—	Badger et al. (2005)
4FKZ	63.0	1.69 Å	*Bacillus subtilis*	Closed	UDP*	UDP-GlcNAc	Structure released in 2013; no associated publication
3OT5	59.2	2.20 Å	*Listeria monocytogenes*	Open	—	—	Structure released in 2010; no associated publication
5ENZ	58.0	1.91 Å	*Staphylococcus aureus*	Closed	UDP*	—	Mann et al. (2016)
1F6D	53.8	2.50 Å	*Escherichia coli*	Partially open	UDP*	—	Campbell et al. (2000)
1VGV	53.8	2.31 Å	*Escherichia coli*	Closed-liganded	UDP-GlcNAc		(Badger et al., 2005) *
3DZC	50.7	2.35 Å	*Vibrio cholerae*	Open	—	—	Structure released in 2008; no associated publication
6VLB	48.1	1.85 Å	*Neisseria meningitidis*	Open	—	—	Hurlburt et al. (2020)
6VLC	48.1	2.15 Å	*Neisseria meningitidis*	Closed	UDP-GlcNAc	—	Hurlburt et al. (2020)
5DLD	47.8	1.45 Å	*Burkholderia vietnamiensis*	Closed	UDP*	UDP-GlcNAc	Structure released in 2015; no associated publication
1V4V	45.4	1.8 Å	*Thermus thermophilus*	Open	—	—	Structure released in 2003; no associated publication
4NEQ	32.1	2.85 Å	*Methanocaldococcus jannaschii*	Open	—	—	Chen et al. (2014)
4NES	32.1	1.42 Å	*Methanocaldococcus jannaschii*	Closed	UDP*	UDP-GlcNAc	Chen et al. (2014)
4HWG	29.5	2.00 Å	*Rickettsia bellii*	Open	—	—	Structure released in 2003; no associated publication
7VYY	38.9	2.44 Å	*Streptomyces kasugaensis*	Open			Rattinam et al. (2022)
7VZA	38.9	2.58 Å	*Streptomyces kasugaensis*	Closed	UDP^*^		Rattinam et al. (2022)
7VZ6	38.9	2.09 Å	*Streptomyces kasugaensis*	Closed	UDP-Glc		Rattinam et al. (2022)

* Only the UDP component of UDP-GlcNAc substrate was modeled in the catalytic site of the indicated structures.

**FIGURE 1:**
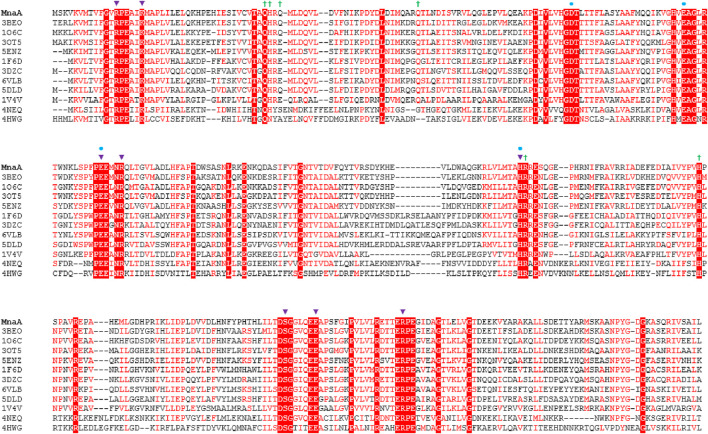
Amino acid sequence alignment of non-hydrolyzing bacterial UDP-GlcNAc 2-epimerases currently published in the PDB (compare with Table 1), including *Pa*MnaA (this study), showing conservation of catalytic and allosteric-site residues in *Pa*MnaA. Red shading indicates residues that are 100% conserved across all epimerases while red font indicates highly conserved residues. The putative catalytic residues are marked with blue circles above the sequence, residues that bind the UDP-GlcNAc substrate are marked with purple triangles, and residues that bind the UDP-GlcNAc allosteric effector are marked with green crosses. 3BEO (*Bacillus anthracis*), 1O6C (*Bacillus subtilis*), 3OT5 (*Listeria monocytogenes*), 5ENZ (*Staphylococcus aureus*), 1F6D (*E. coli*), 3DZC (*Vibrio cholerae*), 6VLB (*Neisseria meningitidis*), 5DLD (*Bacillus vietnamiensis*), 1V4V (*Thermus thermophilus*), 4NEQ (*Methanococcus jannaschii*), 4HWG (*Rickettsia bellii* RML369-C) (Legg, 2022).

**FIGURE 2:**
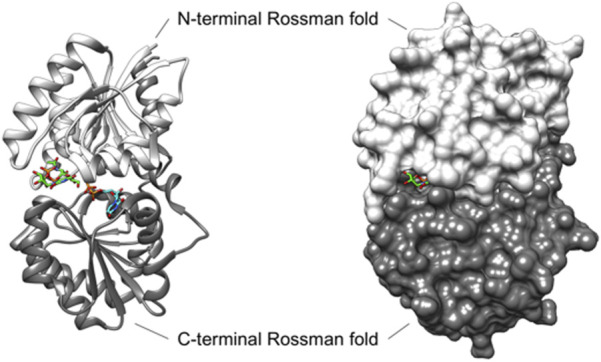
Ribbon (*left*) and surface (*right*) depiction of the UDP-GlcNAc 2-epimerase from *Bacillus anthracis* (PDB ID: 3BEO, Chain A). The enzyme adopts a closed conformation when UDP-GlcNAc (carbon atoms in green) is bound at the allosteric site, and UDP (carbon atoms in blue) is present in the catalytic site (Velloso et al., 2008). In this closed conformation, *Ba*MnaA largely blocks solvent access to the active site, with UDP-GlcNAc bound to the allosteric site further obstructing the small portion of active site that is exposed to the solvent. Although the 3BEO protein model was co-crystallized with the UDP-GlcNAc substrate, no sugar is observed in the catalytic site (Velloso et al., 2008), and an open region is visible where the sugar is likely to bind (Legg, 2022).

Several reported non-hydrolyzing bacterial UDP-GlcNAc 2-epimerases are allosterically activated by their substrate UDP-GlcNAc. In the absence of this activator, virtually no UDP-ManNAc is epimerized in the reverse reaction (Velloso et al., 2008; Mann et al., 2016; Morgan et al., 1997; Kawamura et al., 1978). However, with the addition of only a small amount of UDP-GlcNAc, the reaction reaches equilibrium, as demonstrated with the MnaA orthologs of *B. anthracis* (*Ba*MnaA) and *S. aureus* (*Sa*MnaA) (Velloso et al., 2008; Mann et al., 2016). In contrast, the UDP-GlcNAc 2-epimerase SacA from *N. meningitidis* (*Nm*SacA) does not require UDP-GlcNAc as an allosteric activator, despite the conservation of the allosteric binding site adjacent to the active site (Velloso et al., 2008; Chen et al., 2014). Notably, UDP-GlcNAc was not observed at the allosteric site in a co-crystal structure with *Nm*SacA (Hurlburt et al., 2020). The allosteric site of UDP-GlcNAc 2-epimerases is comprised of six amino acid residues, with variations occurring in the third amino acid residue, which is either glutamine or glutamate in most enzymes (Figure 1). Binding of UDP-GlcNAc to the allosteric site is reported to cause a conformational shift that closes the two Rossman-like domains above the active site, and the UDP-GlcNAc in the allosteric site also directly interacts with the UDP-GlcNAc bound to the catalytic site, preventing access to solvent (Figure 2) (Velloso et al., 2008). The obstruction of the active site is crucial because the formation of the 2-acetoamidoglucal intermediate and UDP is thermodynamically favored over the formation of both UDP-GlcNAc and UDP-ManNAc products. Thus, this more stable intermediate must be trapped within the enzyme to prevent it from becoming the major product (Velloso et al., 2008; Tanner, 2002).

Although several structures of bacterial non-hydrolyzing UDP-GlcNAc 2-epimerases with bound UDP-GlcNAc have been published-*e.g*., in (Velloso et al., 2008; Mann et al., 2016; Chen et al., 2016)-in each case, only UDP-GlcNAc is observed at the allosteric site, while UDP is bound at the catalytic site with the sugar not observed (Figure 2). Comparison with the epimerase structure from *E. coli* reveals that the interactions with UDP in the catalytic site of *Ba*MnaA are conserved (Velloso et al., 2008). However, the residues responsible for proton abstraction/addition have yet to be unequivocally determined (Velloso et al., 2008). Mutations in the region where the GlcNAc moiety of the substrate would be positioned have identified D95 and E131 of the *E. coli* epimerase as candidate residues for proton abstraction, while E117 is suggested to be involved in the second step of the reaction (Velloso et al., 2008). Notably, these residues are conserved in *Ba*MnaA as D100, E136, and E122 (Velloso et al., 2008), and as D99, E135, and E121 in *Pa*MnaA (this study), as well as in other UDP-GlcNAc 2-epimerases (Figure 1).

Crystallography on recombinant *Pa*MnaA conducted in this study yielded an unliganded structure in 2.2-Å resolution, confirming the predicted catalytic and allosteric site of the enzyme. We compiled biochemical and kinetic data of the wild-type enzyme and rational variants, and performed experiments on allosteric activation with the substrate UDP-GlcNAc as well as inhibition with the substrate analog tunicamycin. Along with docking studies, this allowed for a clear picture of the role of the allosteric site and the inhibition mechanism by tunicamycin. Notably, UDP-GlcNAc is not essential for allosteric activation of *Pa*MnaA, but the reverse reaction benefits from its provision. This finding, compared to *Pa*MnaA’s lack of inhibition by tunicamycin, provides valuable insights into the 2-epimerization process.

## 2 Materials and methods

### 2.1 Overexpression of MnaA and mutants

Recombinant *Pa*MnaA epimerase (MnaA.His_6_), encompassing amino acids 1–384 with a Leu-Glu linker followed by a C-terminal His_6_-tag, was expressed from a pET22b (+) (Novagen, Merck, Vienna, Austria)-based construct in *E. coli* BL21 (DE3) cells as described previously (Hager et al., 2018). Site directed mutagenesis at predicted catalytic and allosteric sites of *Pa*MnaA, including Q42A, Q69A, E135A and H241A, was performed following the method described by Blacker and colleagues (Blackler et al., 2018) using oligonucleotides listed in Table 3 and pET22b (+) vector for cloning.

Overexpression of wild-type *Pa*MnaA and variants at 37^o^C with 200 rpm shaking in selective LB broth (10 g L^-1^ tryptone, 5 g L^-1^ yeast extract, 10 g L^-1^ NaCl) supplemented with 100 μg mL^-1^ ampicillin was induced at the mid-exponential growth phase (OD_600_ ∼0.6) by adding isopropyl-β-d-thiogalactopyranoside (IPTG) to a final concentration of 0.6 mM. The cells were incubated for an additional 4 h after IPTG induction and subsequently harvested by centrifugation at 6,500 rpm for 25 min using a Beckmann JA-14 rotor (Beckman Coulter, Brea, CA, United States).

### 2.2 Enzyme purification

For X-ray crystallography, the cell pellet obtained from a 3-L *E. coli* BL21 (DE3) *Pa*MnaA overexpression culture was resuspended in 100 mL of lysis buffer (5% (v/v) glycerol, 20 mM imidazole, 1 M NaCl, 0.2 M arginine, 50 mM NaH_2_PO_4_, pH 7.4), supplemented with a SIGMAFAST™ Protease Inhibitor Tablet (Sigma-Aldrich, St. Louis, MO, United States), 1 mL of 17 mg mL^-1^ phenylmethylsulphonyl fluoride (PMSF) in isopropanol, 2 mM tris(2-carboxyethyl) phosphine (TCEP), and 3 mg of DNase I. The resuspended cells were disrupted using a Misonix Sonicator 3,000 (5 min total time, 10 s on, 10 s off, initial power output 6). After sonication, Triton® X-100 was added to a final concentration of 0.1% (v/v). The cell lysate was then centrifuged at 6,500 rpm for 25 min using a Beckmann JA-17 rotor (Beckman Coulter, Brea, CA, United States); the resultant supernatant was removed and centrifuged again at 10,000 rpm for 60 min to further remove cell debris. The final supernatant fraction (cell crude extract) was incubated for 30 min at 4°C with 3 mL of Thermo Scientific™ HisPur™ Ni-NTA resin (Thermo Fisher Scientific, Waltham, MA, United States) pre-equilibrated in lysis buffer. The lysate-Ni-NTA mixture was then loaded onto a column and washed with 50 mL of wash buffer (5% (v/v) glycerol, 25 mM imidazole, 1 M NaCl, 50 mM NaH_2_PO_4_, 2 mM TCEP, pH 7.4). Recombinant *Pa*MnaA protein was eluted using a gradient of wash buffer (50 mL) and elution buffer (50 mL; 5% (v/v) glycerol, 250 mM imidazole, 1 M NaCl, 50 mM NaH_2_PO_4_, 2 mM TCEP, pH 7.4), and collected at a flow rate of 1 mL min^-1^ in 1.5-mL fractions. Fractions were analyzed for purity by SDS-PAGE gel (12.5% polyacrylamide gel) stained with Coomassie Brilliant Blue G250 (CBB). Those fractions determined to be of the highest purity were pooled.

For enzyme assays, *Pa*MnaA wild-type and mutants were purified from 500-mL batches of *E. coli* BL21 (DE3) *Pa*MnaA-overexpression cultures according to Hager and colleagues (Hager et al., 2018) immediately before use.

### 2.3 MnaA crystallization

The recombinant, purified *Pa*MnaA sample was dialyzed in 4 L of dialysis buffer (20 mM HEPES, pH 7.5) for 4 h at 4°C with gentle stirring, using 3.5-K MWCO Thermo Scientific™ SnakeSkin™ dialysis tubing (Thermo Fisher Scientific, Waltham, MA, United States). The dialyzed sample was then concentrated to 17 mg mL^-1^ in a 3-K MWCO Pall Macrosep® Advance Centrifugal Device (Pall Corporation, Port Washington, NY, United States) by centrifugation at 5,000 rpm in a JA-17 rotor kept at 4°C. Protein concentration was measured at A_280_ using a NanoDrop ND-1000 spectrophotometer (Thermo Fisher Scientific, Waltham, MA, United States).

The concentrated *Pa*MnaA sample was filtered through a 0.22-μm centrifugal filter immediately before setting up the crystallization conditions. Crystal screens of recombinant purified *Pa*MnaA (17 mg/mL) were prepared using an Art Robbins Instrument Crystal Gryphon robot and Hampton 96-well Intelli-Plates. The crystallization plates were stored at 18°C. Initial crystals of *Pa*MnaA were obtained from PEGs II condition number 36 (0.2 M CaCl_2_, 0.1 M Tris/HCl pH 8.5, 20% (w/v) PEG 4000) 1 week after plating. This condition was further optimized by raising the pH and adjusting the concentrations of CaCl_2_ and PEG 4000 (optimized condition: 0.16 M CaCl_2_, 0.1 M Tris/HCl pH 8.8, and 15% PEG 4000). Larger crystallization drops were also prepared (1.5 μL mother liquor to 1.5 μL 17 mL^-1^ MnaA) using hanging drop vapor diffusion at 18°C to generate larger, higher-quality crystals for use in subsequent X-ray diffraction experiments and data collection.

For MnaA crystal ligand soaking, MnaA crystals grown by hanging drop vapor diffusion in optimized PEGs II condition 36 were soaked in mother liquor solution with addition of either 10 mM or 50 mM UDP-α-d-GlcNAc (Sigma-Aldrich, St. Louis, MO, United States) overnight at 18°C.

### 2.4 Data collection, structure solution, and refinement

A crystal obtained by hanging drop vapor diffusion from the optimization of PEGs II condition 36 was resuspended in a mother liquor solution containing 20% MPD for 10–20 s before being flash-frozen at 100 K for data collection. X-ray diffraction data were collected using a Rigaku MicroMax-007HF X-ray generator coupled to a Rigaku Dectris Pilatus3R 200K-A detector. The data were scaled, averaged, and integrated using HKL 2000 (Otwinowski and Minor, 1997). The data set was solved by molecular replacement with a model of *Pa*MnaA predicted by Phyre2 (Kelley et al., 2015) and PHASER from the CCP4 program suite (Winn et al., 2011; McCoy et al., 2007). All subsequent model building and refinement steps were carried out using Coot (Emsley et al., 2010) and Refmac5 from the CCP4 suite (Winn et al., 2011; Murshudov et al., 2011).

### 2.5 Activity measurement of *Pa*MnaA

Epimerization catalyzed by *Pa*MnaA was measured by RP-HPLC (U3000, Dionex) run in 0.4 M sodium phosphate buffer, pH 6.4, with UV detection at 254 nm using a Hyperclone 5 µm 120 A 150 × 4.6 mm (Phenomenex) column at a flow rate of 0.6 mL min^-1^ (Hager et al., 2018). The reaction set-up utilized a 10 mM sodium phosphate buffer, pH 8.0, as recommended by (Mann et al., 2016), with an assay volume of 50 µL. To inactivate the enzyme, the reactions were heated for 1 min at 100°C and then centrifuged at 10,000 g, 5 min at room temperature (RT). Various reaction conditions were evaluated in the forward direction with 0.5 mM UDP-GlcNAc (Sigma) and 420 ng of *Pa*MnaA, including temperature (4°C, RT, 30°C and 37°C), pH (7.0, 7.5, 8.0, 8.5), and salt additions (MgCl_2_, NaCl, MgSO_4_, CaCl_2_, MnSO_4_; 50 and 10 mM, each) with a 5-min incubation time. The reverse reaction was investigated to test UDP-GlcNAc as an allosteric activator (1%, 5% and 10%) using 0.1 mM UDP-ManNAc [chemically synthesized as described in (Hager et al., 2018)] and 420 ng of *Pa*MnaA with a 45-min incubation time.

A reaction time course was recorded to determine initial rates. For the forward reaction (0.1 mM UDP-GlcNAc and 50 ng of *Pa*MnaA), aliquots were taken at 1, 3, 5, 8, 10 and 20 min. For the reverse reaction (0.1 mM UDP-ManNAc and 210 ng of *Pa*MnaA), aliquots were taken at 10, 30, 60, 75, 90, 105 and 120 min.

Tunicamycin was dissolved in DMSO at a concentration of 1 mM and tested as a potential inhibitor of the reaction at concentrations of 0.5 µM, 1 μM, 10 μM, 50 μM, 100 µM and 200 μM, corresponding to a substrate-to-inhibitor ratio of 1:0.005 (0.5 µM), 1:0.01 (0.1 µM), 1:0.1 (10 µM), 1:0.5 (50 µM), 1:1 (100 µM), and 1:2 (200 µM), respectively (Mann et al., 2016), with a 2-h incubation time and a pre-incubation of tunicamycin with the enzyme for 15 min. To test whether DMSO affects the epimerization reaction, a control reaction was done by adding DMSO without tunicamycin.

### 2.6 Enzyme kinetics

The enzyme assays were performed in a total volume of 50 μL sodium phosphate buffer (10 mM, pH 8.0) containing 50 mM MgCl_2_. UDP-GlcNAc and UDP-ManNAc were varied at concentrations of 0.1, 0.25, 0.5, 0.75, 1, 1.25, 1.5, 1.75, 2, 3, 4, 5, 7.5 and 10 mM, and at 0.083, 0.125, 0.250, 0.375, 0.5, 0.75, 1, and 2 mM, respectively, with either 50 ng or 210 ng of *Pa*MnaA. For the *Pa*MnaA variants, 50–1,440 ng of enzyme were used. The reactions were incubated for either for 5 min at 30°C (forward reaction) or 45 min at 30°C (reverse reaction). Upon varying the UDP-ManNAc or UDP-GlcNAc concentrations, apparent kinetic results were calculated by fitting the data (average values of triplicates) into the Michaelis Menten-equation using GraphPad Prism (version 9.1.2; GraphPad, San Diego, CA, United States).

### 2.7 Docking of tunicamycin

To obtain a structural model of the unmodeled residues in chain A and chain B of *Pa*MnaA which correspond to flexible loop regions, SWISS-MODEL (https://swissmodel.expasy.org/) (Waterhouse et al., 2018) was used. For comparability with the other epimerases (*Sa*MnaA and *Nm*SacA), in the subsequent docking studies, additionally a structural homology model of *Pa*MnaA was created using *Sa*MnaA (5ENZ) as a template to obtain a closed conformation. After protein structure modelling, docking studies of *Pa*MnaA chain A in comparison to the crystal structure of *Sa*MnaA (5ENZ), chain A and *Nm*SacA (6VLC), all in a closed conformation, with tunicamycin [ZINC database (Sterling and Irwin, 2015)] as a potential inhibitor were conducted using AutoDockTools 1.5.7. The active/binding site for tunicamycin was predicted by selecting the conserved allosteric residues in *Pa*MnaA (E42, H43, E45, Q69, R209 and H241) as well as in *Nm*SacA (R10, M42, H245, E295), based on the residues in *Sa*MnaA (E39, H40, E66, R206 and H238) (Mann et al., 2016). Furthermore, docking was done over the whole protein of *Pa*MnaA and *Sa*MnaA to identify putative other binding sites which might have escaped detection by docking to the allosteric site. The interactions of the inhibitor with the binding-site residues at the lowest free binding energy was used to create interaction diagrams for tunicamycin docked to *Pa*MnaA, *Sa*MnaA, and *Nm*SacA using MOE (Molecular Operating Environment, v2019). Residues of the inhibitor within a distance of 4.5 Å were considered for interactions.

### 2.8 Visualization and graphics

All figures depicting protein structures and surfaces were created using the UCSF Chimera Extensible Molecular Modeling System, developed by the Resource for Biocomputing, Visualization, and Informatics at the University of California, San Francisco, with support from NIH P41-GM103311 (Pettersen et al., 2004).

## 3 Results

### 3.1 *Pa*MnaA crystal screening and optimization

For X-ray crystallography, purified *Pa*MnaA (Supplementary Figure S2) from a 3-L overexpression culture of *E. coli* BL21 (DE3) cells was used at a concentration of 17 mg mL^-1^. Initial *Pa*MnaA crystal hits were observed in the PEGs II screen condition number 36 (0.2 M CaCl_2_, 0.1 M Tris/HCl pH 8.5, and 20% PEG 4000). This condition was optimized to yield crystals of sufficient quality for use in X-ray diffraction experiments by decreasing the PEG 4000 concentration (Supplementary Figure S3), increasing the crystallization drop size (Supplementary Figure S4) and increasing the pH (Supplementary Figure S5). The final condition, consisting of 0.16 M CaCl_2_, 0.1 M Tris/HCl pH 8.8, and 15% PEG 4000, yielded the final *Pa*MnaA crystal used for data collection and MnaA structure determination (detailed below).

### 3.2 Data collection and structure determination

All data collection and refinement statistics for the unliganded structure of *Pa*MnaA are shown in Table 2. The crystal structure was solved at a final resolution of 2.20 Å in space group *P*12_1_1 with two molecules in the asymmetric unit (AU). A search model generated by Phyre2 (Kelley et al., 2015) was used for molecular replacement in PHASER, part of the CCP4 program suite (Winn et al., 2011). The solution was finalized through iterative refinement and model improvements using REFMAC5 (Winn et al., 2011) and the Coot macromolecular model building software (Emsley et al., 2010). The final structure yielded an average B-factor of 36.1 Å^2^, with R_work_ and R_free_ values of 21.0% and 26.2%, respectively.

**TABLE 2:** Data collection and refinement statistics for MnaA (Legg, 2022).

Protein	MnaA
Data collection	
Space group	P12_1_1
Resolution (Å)	50.00–2.20 (2.28–2.20)
Cell dimensions	
*a*(Å)	45.82
*b*(Å)	81.76
*c*(Å)	107.72
*α*(°)	90
*β*(°)	97.14
*γ*(°)	90
Z	2
*R*_*sym*_	0.057 (0.211)
*R*_*pim*_	0.031 (0.133)
*C/C*_*1/2*_	(0.957)
I/σ(I)	18.3 (4.4)
Completeness (%)	99.0 (91.4)
Redundancy	4.0 (3.0)
Unique reflections	39705
Refinement	
Resolution (Å)	25.41–2.20
No. reflections	37651
*R*_*work*_(%)	21.0
*R*_*free*_(%)	26.3
No. atoms	
Protein	5,658
Ligand	14 (PEG^a^)
Water	183
*B* factors	
Protein	36.1
Ligand	52.5
Water	36.0
Average	36.1
Ramachandran	
Favored (%)	95.6
Allowed (%)	4.4
r.m.s. bonds (Å)	0.0078
r.m.s. angles (°)	1.66

**TABLE 3:** Oligonucleotide primers used during site directed mutagenesis.

Oligonucleotide primers used for PCR amplification		
Mutation (A)	Primer	Sequence (5′-3′)
Q42	Q42A_fwd_3.2	GTA TGC GTC ACG GCC gcg CAT CGT CAG ATG TTG
Q42	Q42A_rev_3.2	CAA CAT CTG ACG ATG cgc GGC CGT GAC GCA TAC
Q69	Q69A_fwd_3.2	TCT GTG CGT GTC CTT gcg GGA CTG GAG CCT GTT
Q69	Q69A_rev_3.2	AAC AGG CTC CAG TCC cgc AAG GAC ACG CAC AGA
E135	E135A_fwd_3.2	CTT TCT CCG TTT CCA gcg GAA ATG AAC CGT CAA
E135	E135A_rev_3.2	TTG ACG GTT CAT TTC cgc TGG AAA CGG AGA AAG
H241	H241_fwd_2.3	ATC GTA TAT CCG GTT gcc CCA AGT CCG GCT GTA
H241	H241_rev_2.3	TAC AGC CGG ACT TGG ggc AAC CGG ATA TAC GAT

**FIGURE 3:**
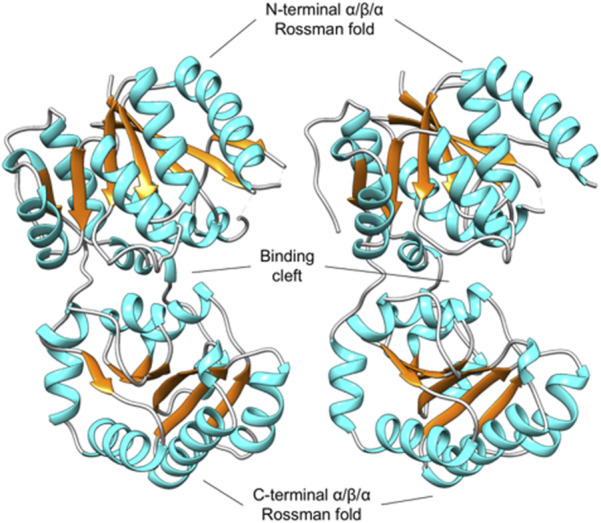
Unliganded, open conformation of MnaA from *Paenibacillus alvei* in the absence of substrate, adopting a GT-B fold. The MnaA crystal structure was solved with two molecules (A and B) in the AU (shown *left* and *right*). The N-terminal and C-terminal domain each adopts a distinct Rossmann fold, characteristic of the GT-B fold. The space between these two folds forms an interdomain cleft that contains the active site (Legg, 2022).

As expected, the overall structure of *Pa*MnaA forms a GT-B fold, with both the N-terminal and C-terminal domains adopting distinct Rossmann folds connected by a short linker region (residues 173–200). The space between these two folds forms an interdomain cleft containing the active site (Figure 3). The N-terminal domain (consisting of residues 2–172) shows good electron density for most residues, except for positions 42–45 and 64–69 in molecule A, and those at positions 43–46 and 66–68 in molecule B, which showed no corresponding electron density and, thus, were not modeled. The N-terminal domain forms an extended β-sheet structure with 3-2-1-4-5-6-7 connectivity between the seven β-strands (β1-β7), which are linked by six α-helices (α1-α6) (Figure 3). The unmodeled residues in molecules A and B correspond to flexible loop regions between the β2 and α2, and β3 and α3 secondary structural elements. The linker region connecting the N- and C-terminal domains (residues 173–200) consists of two α-helices (α7 and α8). Residues in this region mostly show good electron density, apart from residues 186–192 in molecule A, and residues 186–190 in molecule B, which show only partial main chain and side-chain electron density. The C-terminal domain (residues 201–347) exhibits good electron density for all residues in both molecules A and B. Similar to the N-terminal domain, the C-terminal domain forms an extended β-sheet structure (with 3-2-1-4-5-6 connectivity between the β-strands) which comprises six β-strands (β8-β13) linked by five α-helices (α9-α13). Additionally, there are three α-helices (α14-α16) after the final β-strand of the C-terminal domain, with the last helix associated with the N-terminal domain of the *Pa*MnaA crystal structure (Figure 3).

### 3.3 *Pa*MnaA crystal ligand soaking

Co-crystal screening of *Pa*MnaA with UDP-GlcNAc substrate did not produce crystal hits; therefore, MnaA crystals obtained from optimized PEGs II condition number 36 (0.16 M CaCl_2_, 0.1 M Tris/HCl, pH 8.8, 15% PEG 4000) were soaked in a drop of mother liquor supplemented with UDP-α-d-GlcNAc in an attempt to generate a donor-bound structure. Overnight soaks in 10 mM UDP-α-d-GlcNAc produced no change to the resultant crystal structures, with no bound ligand observed (data not shown). Overnight soaking in 50 mM UDP-α-d-GlcNAc caused all crystals to dissolve.

### 3.4 Optimal conditions for *Pa*MnaA activity

Purified, C-terminal His_6_-tagged *Pa*MnaA was obtained at a concentration of 0.42 mg mL^-1^ in 20 mM sodium phosphate buffer, pH 7.5. For enzymatic assays, *Pa*MnaA was used directly after recombinant production.

Using our established HPLC assay (Hager et al., 2018), we evaluated the optimal reaction conditions for measuring the initial rate of *Pa*MnaA catalysis, including temperature, pH, salt supplement, and incubation time and temperature. No clear temperature optimum was identified within the range tested, but a significantly reduced turnover was observed at 4°C (not shown). Therefore, 30°C was chosen as the assay temperature. The reaction was mostly unaffected by pH changes within a range of 7.0–8.5 (Supplementary Figure S6), so a pH of 8.0, as reported in the literature, was used. Interestingly, all salts tested*, i.e*., MgCl_2_, MgSO_4_, and NaCl, resulted in a significantly better performance of the enzyme compared to the reaction without addition of metal ions (Supplementary Figure S7). The addition of 50 mM MgCl_2_ resulted in the best yield and was thus used for the enzyme assays. The optimal incubation time was individually determined for wild-type *Pa*MnaA and the enzyme mutants. For the wild-type enzyme, the initial rate incubation time was determined to be 5 min for the forward reaction and 45 min for the reverse reaction. For the variants, the initial rate incubation time to reach the end of the linear phase of the reaction was determined to be between 5 and 30 min.

### 3.5 Kinetics of *Pa*MnaA

**FIGURE 4:**
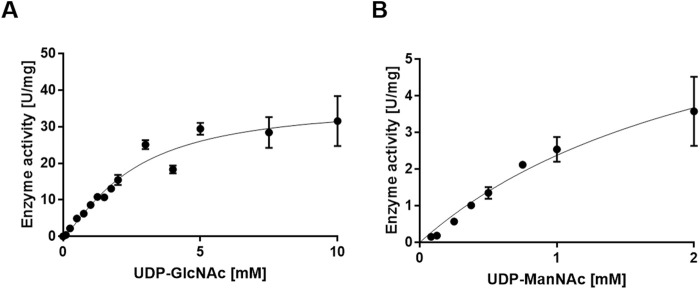
Michaelis-Menten curve for the *Pa*MnaA-catalyzed epimerization of **(A)** UDP-GlcNAc to UDP-ManNAc (forward reaction) and **(B)** UDP-ManNAc to UDP-GlcNAc (reverse reaction).

Using UDP-GlcNAc as a substrate, the apparent *k*_cat_ value of *Pa*MnaA was determined to be 33.44 s^-1^, with a *K*_M_ value of 3.91 mM (Figure 4A; Table 5). When UDP-ManNAc was used as the substrate, the apparent *k*_cat_ value was 6.02 s^-1^, with a *K*_M_ value of 2.41 mM (Figure 4B; Table 5). *Pa*MnaA exhibited an approximately three times higher catalytic efficiency towards UDP-GlcNAc (*k*_cat_/*K*_M_ = 8.56 mM s^-1^) compared to UDP-ManNAc (*k*_cat_/*K*_M_ = 2.49 mM s^-1^). However, *Pa*MnaA showed an approximately two times higher *K*_M_ value for UDP-GlcNAc (3.91 mM) than for UDP-ManNAc (2.41 mM), indicating a lower affinity for UDP-GlcNAc. This lower affinity for UDP-GlcNAc is also seen when compared to other enzymes such as RffE of *E. coli* (Samuel and Tanner, 2004) or *Sa*MnaA (Mann et al., 2016). Notably, a higher affinity for UDP-ManNAc than for UDP-GlcNAc was reported also for *Sa*MnaA (Mann et al., 2016), but not for *Nm*SacA (Hurlburt et al., 2020) (Table 5). In contrast to *Pa*MnaA, *Sa*MnaA shows a *K*_M_ value for UDP-ManNAc that is four times lower than that for UDP-GlcNAc (Mann et al., 2016), whereas for *Pa*MnaA, this difference is only twofold. The determined *K*_M_ of *Pa*MnaA for UDP-GlcNAc is almost doubled compared to that of *Ba*MnaA (2.2 mM) and comparable to that of *Nm*SacA (3.6 mM) (Zhang et al., 2016). Furthermore, the *k*_cat_ value of 33.44 s^-1^ (forward reaction) and 6.02 s^-1^ (reverse reaction) of *Pa*MnaA is comparable to that calculated for *Nm*SacA for UDP-GlcNAc (Zhang et al., 2016), whereas *Ba*MnaA shows a lower *k*_cat_ of around 7.0 s^-1^, comparable to 6.01 s^-1^ determined for the reverse reaction of *Pa*MnaA.

Overall, *Pa*MnaA exhibited a lower affinity to UDP-GlcNAc in the forward reaction but an increased turnover and catalytic efficiency; consequently, the increased turnover and catalytic efficiency compensates for the lower affinity and leads to the previously determined 1:9-ratio in the UDP-ManNAc epimerization reaction (Hager et al., 2018).

Two of the *Pa*MnaA variant constructs, Q42A and H241A, which targeted the predicted allosteric site (Figure 1), revealed a significant reduction of the *k*_cat_ value (0.05 s^-1^ and 0.53 s^-1^, respectively) and a 2.5-fold lower *K*_M_ value (1.61 mM) for Q42A and a similar *K*_M_ value for H241A (3.27 mM) (Supplementary Figures S8, S9). Both mutations showed a pronounced influence on the *k*_cat_ contrary to the corresponding *Ba*MnaA variants, where the influence was not as pronounced (Velloso et al., 2008). Another point mutation within the allosteric site of *Pa*MnaA, Q69A, showed a 2.3-fold decrease in affinity to UDP-GlcNAc (*K*_M_ of 8.92 mM) and a similar *k*_cat_ value of 37.92 s^−1^) compared to the one of the wild-type enzyme (Supplementary Figure S10). Thus, intervention at the allosteric site had only a small effect on the affinity of the enzyme for UDP-GlcNAc. Conversely, the variants of the homologous residues in *Ba*MnaA targeting the allosteric site showed a more pronounced effect; the *K*_M_ values showed a 5- to 8-fold increase, resulting in significantly poorer affinity to the substrate (Velloso et al., 2008) (Table 5). *Pa*MnaA variant E135A was a direct intervention on a putative catalytic residue and led to a drastic reduction in activity and no kinetic data could be obtained.

### 3.6 *Pa*MnaA activity is independent of allosteric activation

**FIGURE 5:**
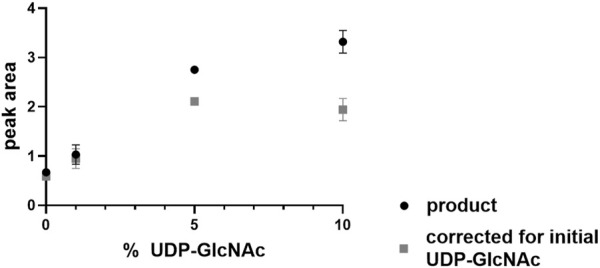
UDP-ManNAc product peak area increase during *Pa*MnaA catalysis upon provision of 1%, 5% and 10% of UDP-GlcNAc at 5 min of incubation.

Next, it was investigated whether UDP-GlcNAc serves as an allosteric activator of the *Pa*MnaA-catalyzed epimerization reaction (reverse reaction), as reported for some UDP-GlcNAc 2-epimerases of other bacteria. We found that, while the reverse reaction did not require addition of UDP-GlcNAc to the assay mixture, the reaction rate was doubled after 5 min when 5% UDP-GlcNAc was included in the reaction mixture. Notably, provision of UDP-GlcNAc at 10% only led to a slight increase of the reaction rate with a bad cost-to-benefit ratio (Figure 5). Thus, *Pa*MnaA shows a similar behavior regarding allosteric activation as the *Nm*SacA enzyme, where UDP-GlcNAc is not necessary for UDP-ManNAc epimerization but enhances the reaction rate, if provided (Hurlburt et al., 2020). Modelling the activity curve using the Hill-equation revealed a Hill-coefficient of 1.31 which is lower as compared to the one of *E. coli* epimerase (1.81) (Samuel and Tanner, 2004) and therefore supports a lower degree of allosteric control of *Pa*MnaA.

### 3.7 Tunicamycin does not inhibit *Pa*MnaA

**FIGURE 6:**
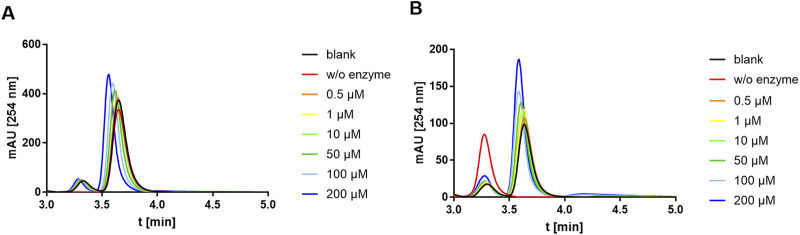
HPLC chromatograms recorded during addition of tunicamycin to the *Pa*MnaA-catalyzed **(A)** forward reaction (UDP-GlcNAc to UDP-ManNAc) and **(B)** reverse reaction (UDP-ManNAc to UDP-GlcNAc). Chromatograms with 0.5 µM (orange), 1 µM (yellow), 10 µM (light green), 50 µM (dark green), 100 µM (light blue) and 200 µM (dark blue) tunicamycin, in comparison to the blank (black) without addition of tunicamycin, are shown. UDP-ManNAc and UDP-GlcNAc can be clearly discerned based on their retention time at ∼3.3 min and ∼3.7 min, respectively. The shift to the left with increasing tunicamycin concentration is due to the presence of a gradually increasing amount of DMSO.

Based on the study of *Sa*MnaA, demonstrating a dose-dependent impact of tunicamycin on the enzyme reaction (Mann et al., 2016), the impact of tunicamycin on the *Pa*MnaA-catalyzed reaction(s) was investigated. Specifically, tunicamycin was tested at concentrations of 0.5 µM, 1 μM, 10µM, 50µM, 100 μM and 200 µM in forward and reverse reaction. However, no inhibition of *Pa*MnaA by tunicamycin was seen in either direction of the reaction, as shown in Figure 6A (forward reaction) and Figure 6B (reverse reaction), respectively, regardless of the presence of tunicamycin at low or high concentration.

### 3.8 Docking of tunicamycin to *Pa*MnaA compared to the homolog of *Staphylococcus aureus* and *Neisseria meningitidis*

**FIGURE 7:**
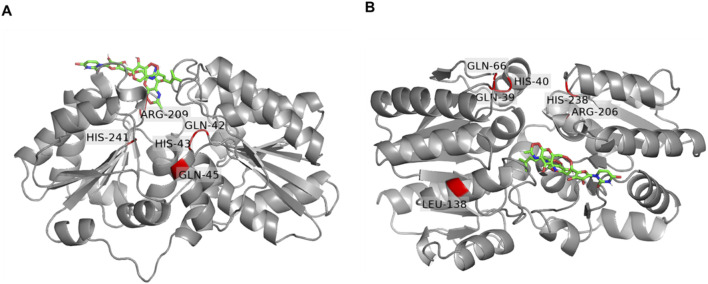
Docking of tunicamycin (green) to the UDP-GlcNAc 2-epimerase of *Paenibacillus alvei* compared to *Staphylococcus aureus*. **(A)** *Pa*MnaA molecule A modeled by Swiss Modell with docked tunicamycin into the allosteric site. Conserved residues (Q42, H43, Q45, Q69, R209 and H241) are highlighted in red; **(B)** *Sa*MnaA molecule A (5ENZ) with docked tunicamycin into the allosteric site with conserved residues (Q39, H03, Q66, R206 and H238) highlighted in red. Residues were visualized by PyMoL (Open Source Version 2.4; https://github.com/schrodinger/pymol-open-source).

Based on the observed lack of inhibition of *Pa*MnaA by tunicamycin (Figure 6A, B) and the absence of a requirement for allosteric activation (Figure 5), we sought to gain insight into the molecular mechanism of tunicamycin resistance of *Pa*MnaA through molecular docking of the antibiotic to the enzyme’s allosteric site. A complete structural homology model of molecule A was created by SWISS-MODEL using the 5ENZ structure (*Sa*MnaA) as a template to enforce a closed conformation of *Pa*MnaA, for comparability with literature data, and additionally accounting for the missing residues within the crystal structure of *Pa*MnaA. Subsequently, tunicamycin docking was performed into the predicted allosteric site of *Pa*MnaA (Figure 7A) and compared to a similar study on *Sa*MnaA (5ENZ) chain A (Figure 7B), which had previously shown to be inhibited by the antibiotic. The docking studies were conducted using AutodockTools. For comparison, the antibiotic was also docked into a model of *Nm*SacA, since it shows a similar behavior towards the allosteric activator (Hurlburt et al., 2020) as *Pa*MnaA, although there is no data in the literature on the impact of tunicamycin on *Nm*SacA (Supplementary Figure S11). The position of tunicamycin in *Pa*MnaA, *Sa*MnaA and *Nm*SacA differs regarding the spatial orientation of the antibiotic’s side chains. Notably, the opening angle defined as the angle between the Cα atoms of the residues 131, 216, 352 in *Pa*MnaA crystal structure was found to be 45°, while that in the *Pa*MnaA_closed model is 31° which agrees with 31° and 32° in the crystal structure of *Sa*MnaA and *Nm*SacA, respectively (de Azevedo and Nascimento, 2019).

**FIGURE 8:**
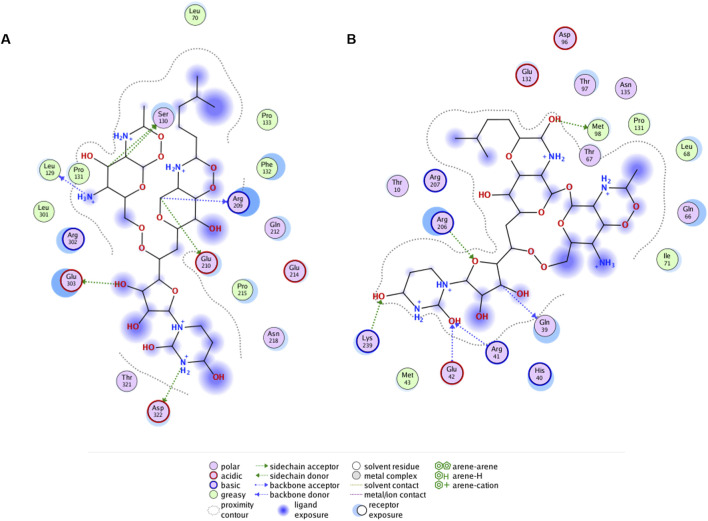
Ligand interaction diagrams for tunicamycin docked to predicted allosteric site residues of **(A)** *Pa*MnaA (SWISS Model), **(B)** *Sa*MnaA (5ENZ). Interacting residues of the enzymes are within a distance of 4.5 Å to the antibiotic. Interaction diagrams were calculated in MOE (Molecular Operating Environment, v2019).

Ligand interaction diagrams did not reveal prominent interactions between that part within tunicamycin that is analogous to UDP-GlcNAc and any of the indicated allosteric site residues in *Pa*MnaA (Figure 8A). This contrasts with the results obtained for *Sa*MnaA (Figure 8B), where two of the indicated allosteric site residues, *i.e.*, Q39, corresponding to Q42 in *Pa*MnaA (Q41 in *Nm*SacA) as well as R206, corresponding to R209 in *Pa*MnaA (R213 in *Nm*SacA), showed interactions with the inhibitor. Both of these residues localize within the allosteric site. In comparison, the docking of tunicamycin to *Pa*MnaA (Figure 8A), revealed the residues L129, S130, R209, E210, E303 and D322 as potential interaction partners with the inhibitor. Out of these residues, only R209 is present within the predicted allosteric or substrate binding site of the enzyme. However, no interaction of this amino acid with the UDP-GlcNAc-analogous portion of the inhibitor were revealed in the ligand interaction diagram, explaining that the activity of *Pa*MnaA remained unaffected by both low and high tunicamycin concentrations. Additionally, docking over the whole protein of *Pa*MnaA (Supplementary Figure S12A) and *Sa*MnaA (Supplementary Figure S12B) was performed. The docking of tunicamycin over *Sa*MnaA resulted in similar predicted interaction partners as those revealed by docking to the allosteric site, whereas in *Pa*MnaA, the antibiotic was docked far away from the predicted allosteric or substrate binding site of the enzyme, underlaying the failure of inhibition by the antibiotic. In the case of *Nm*SacA (Supplementary Figure S11B), residues R10 and E295 present in the substrate binding site were revealed to be exposed to tunicamycin, as well as M42 and H245, of which neither is located close to the allosteric or substrate binding site.

## 4 Discussion

The bacterial cell wall is crucial for bacterial physiology and fitness. The involvement of non-hydrolyzing UDP-GlcNAc 2-epimerases in the cell wall biosynthesis of several Gram-negative (Hurlburt et al., 2020; Morgan et al., 1997) and Gram-positive (Velloso et al., 2008; Mann et al., 2016; Kawamura et al., 1978) pathogens suggests that this enzyme family could serve as a novel antimicrobial target.

To better understand the epimerization reaction mechanism of *Pa*MnaA and its apparent lack of requirement for UDP-GlcNAc for activity (this study), as well as to determine the structural basis of its tunicamycin resistance (this study), we analyzed the reaction behavior of wild-type *Pa*MnaA and rational enzyme variants and we solved the ligand-free crystal structure of *Pa*MnaA (Figure 3). Using molecular docking, we then compared the residues in the enzyme’s predicted allosteric site with the situation in *Sa*MnaA (Supplementary Figure S12), which was previously shown to be inhibited by the antibiotic, but otherwise shows apparent similarities to *Pa*MnaA in its reaction behavior (Mann et al., 2016). Additionally, we have included in the docking study *Nm*SacA, which, like *Pa*MnaA, does not require allosteric activation by UDP-GlcNAc (Hurlburt et al., 2020).

UDP-GlcNAc 2-epimerases typically convert UDP-GlcNAc to UDP-ManNAc at a 9:1-ratio (forward reaction) to fuel the biosynthesis routes of various cell wall polymers. All investigated enzymes, except for *Nm*SacA (Hurlburt et al., 2020) and *Pa*MnaA (this study), are strictly allosterically regulated, requiring UDP-GlcNAc to catalyze the epimerization of UDP-ManNAc to UDP-GlcNAc (reverse reaction). In the absence of UDP-GlcNAc, the epimerases were shown to fail in the epimerization of UDP-ManNAc, but UDP-GlcNAc alone can be epimerized readily to form UDP-ManNAc until equilibrium is reached.

The *P*aMnaA structure contains two molecules in the crystallographic asymmetric unit, both displaying nearly identical conformations with an overlap Cα rmsd of 0.32 Å for 370 equivalent residues. Each monomer exhibits a GT-B fold, where the N- and C-termin (Figure 3). This arrangement aligns with the architecture of previously published structures of other bacterial non-hydrolyzing UDP-GlcNAc 2-epimerases (1, 4, 5, 28, 41, 42) (Table 1), where substrate binding occurs in the cleft located between the two domains (1, 4, 5, 28, 42). In the absence of substrate, *Pa*MnaA adopts an unliganded conformation corresponding to the “open” state observed for these other enzymes (Hurlburt et al., 2020; Badger et al., 2005; Chen et al., 2014), where the inner surfaces of the binding cleft are exposed to solvent. This open state is believed to facilitate substrate entry and binding within the cleft (Mann et al., 2016; Campbell et al., 2000). In previously published UDP-GlcNAc 2-epimerase structures, substrate binding induces a significant conformation change in which the two Rossmann-like domains “clamp down” on the bound substrate, allowing the epimerization reaction to proceed in the “closed” enzyme state (Velloso et al., 2008; Mann et al., 2016; Campbell et al., 2000). Key residues involved in substrate binding in other UDP-GlcNAc 2-epimerases are conserved in *Pa*MnaA (R13, L18, E135, R139, H208, S285, and E291) (Figure 1; Table 4). Additionally, residues D99, E121, E135 and H208 of the putative catalytic site (Figures 4, 5; Table 4) are conserved in the *Pa*MnaA amino acid sequence. Thus, despite variations in the overall amino acid sequences of these enzymes (ranging from about 66.2% to 29.5% sequence identity), the residues crucial for catalysis and substrate binding appear to be conserved.

**TABLE 4:** Allosteric/catalytic site residues in *Paenibacillus alvei* MnaA based on homology with other UDP-GlcNAc 2-epimerases (Legg, 2022) (see Table 1).

Substrate binding site residues	Putative catalytic residues	Allosteric site residues
Arg13	Asp99	Gln42^*^
Lys18	Glu121	His43^*^
Glu135	Glu135	Gln45^*^
Arg139	His208	Gln69^*^
His208		Arg209
Ser285		His241
Glu291		

^*^Residues indicated by an asterisk were not modelled in the crystal structure, but with SWISS model.

Some bacterial non-hydrolyzing UDP-GlcNAc 2-epimerases function *via* allosteric regulation. Crystal structures of UDP-GlcNAc 2-epimerases from Gram-positive *B. anthracis* (Velloso et al., 2008) and *S. aureus* (Mann et al., 2016), as well as the archaeon *Methanococcus jannaschii* (Chen et al., 2014), reveal that this allosteric regulation occurs through the binding of the UDP-GlcNAc substrate in a conserved site next to the active site. In these structures, the active site contains only a bound UDP molecule. This is due to hydrolysis of the GlcNAc residue when enzyme is soaked or co-crystallized with its natural substrate, UDP-GlcNAc (Velloso et al., 2008; Chen et al., 2014).

**TABLE 5:** Comparison of the kinetic data obtained for the forward reaction (fwd) catalyzed by the *Pa*MnA wild-type enzyme and allosteric variants of *Pa*MnaA with those from *Bacillus anthracis* (Velloso et al., 2008), *Neisseria meningitidis* (Zhang et al., 2016), and *Staphylococcus aureus* (Mann et al., 2016) wild-type UDP-GlcNAc 2-epimerases (fwd).

*Paenibacillus alvei*				*Bacillus anthracis*				*Neisseria meningitidis*				*Staphylococcus aureus*			
This study				Velloso et al., 2008				Zhang et al., 2016				Mann et al., 2016			
	*K*_*M_fwd*_[mM]	*k*_*cat_fwd*_[s^-1^]	*k*_*ca*t_/*K*_*M*_[s^-1^mM^-1^]		*K*_*M_fwd*_[mM]	*k*_*cat_fwd*_[s^-1^]	*k*_*ca*t_/*K*_*M*_[s^-1^mM^-1^]		*K*_*M_fwd*_[mM]	*k*_*cat_fwd*_[s^-1^]	*k*_*ca*t_/*K*_*M*_[ s^-1^mM^-1^]		*K*_*M_fwd*_[mM]	*k*_*cat_fwd*_[s^-1^]	*k*_*ca*t_/*K*_*M*_[ s^-1^mM^-1^]
Wild-type	3.91	33.44	8.56	Wild-type	2.20	7.90	3.60	Wild-type	3.60	31.00	8.60	Wild-type	0.41	-	-
Q42A	1.61	0.53	0.03	Q43A	17.20	1.20	0.07								
Q69A	8.92	37.92	4.25	Q70A	11.10	2.06	0.19								
H241A	3.27	0.53	0.16	H242A	10.40	0.22	0.021								

One goal of this study was to obtain MnaA co-crystal structures in complex with the UDP-GlcNAc substrate. This structure would verify whether *Pa*MnaA undergoes the same conformational change upon UDP-GlcNAc binding to the allosteric site as observed in other published UDP-GlcNAc 2-epimerase structures (Velloso et al., 2008; Mann et al., 2016; Chen et al., 2016). Unfortunately, co-crystallization of MnaA and UDP-GlcNAc has not yielded crystals. The next strategy involved soaking experiments using crystals obtained from the optimized unliganded MnaA crystallization condition. Most soaking trials shattered the crystal and did not yield specimens amenable to X-ray diffraction. If *Pa*MnaA substrate- and effector-binding is at all analogous to that of *Ba*MnaA, crystal dissolution could be due to UDP-GlcNAc binding to the allosteric site and a concomitant conformational change in the protein (open *versus* closed conformation). While this theory is likely based on known UDP-GlcNAc 2-epimerase structures, it remains uncertain and awaits characterization of an intact, substrate-bound *Pa*MnaA structure. UDP-GlcNAc binding at the allosteric site likely functions to exclude solvent from the active site and optimize the enzyme’s catalytic conformation (Velloso et al., 2008; Mann et al., 2016; Campbell et al., 2000; Chen et al., 2014). Additionally, the GlcNAc moiety of the allosteric effector is proximal to the active site and is poised to make extensive interactions with the bound UDP molecule (Velloso et al., 2008; Mann et al., 2016; Chen et al., 2014). In these enzymes, the observed allosteric site residues are not uniformly conserved, but their chemical identities are generally preserved (*e.g.*, interchange of polar residues glutamine or serine) (Figure 1). Notably, *Pa*MnaA shows the greatest level of amino acid sequence identity (67.2%) with *Ba*MnaA (Table 1); the enzymes share the same five allosteric residues (Figure 1; Table 4). Therefore, contact with UDP-GlcNAc at this site may similarly impact the binding and catalysis of the *P. alvei* enzyme. Velloso *et al.* (2008) hypothesized that the binding of the allosteric effector prevents the thermodynamically more stable reaction intermediates, UDP and 2-acetoamidoglucal, from diffusing away from the catalytic site during the epimerization reaction (Velloso et al., 2008). Furthermore, binding at the allosteric site may shield the catalytic site from solvent in the closed catalytic state of the enzyme, thereby preventing unproductive reactions between water and bound substrate (Velloso et al., 2008; Mann et al., 2016; Chen et al., 2014). However, currently, there is no structural insight into how the less-stable UDP-GlcNAc or UDP-ManNAc substrates are able to depart the catalytic site, while the more-stable 2-acetoamidoglucal and UDP intermediates remain (Velloso et al., 2008).

According to current knowledge, the allosteric site of UDP-GlcNAc 2-epimerases exclusively and specifically binds UDP-GlcNAc to induce a conformational shift that activates the enzyme. When UDP-ManNAc alone is available, the enzyme remains inactive (Velloso et al., 2008). This inactivity occurs either because UDP-ManNAc does not bind to the allosteric site or, if it does bind, it does not induce the conformational shift necessary for enzyme activation. In contrast, the catalytic site does not discriminate between the epimers and can interconvert UDP-GlcNAc and UDP-ManNAc, implicating that it recognizes both (Velloso et al., 2008; Tanner, 2002). A different scenario might be that the two domains of the epimerase are in a dynamic equilibrium between an open and closed conformation. The equilibrium between these conformational states could be modulated by an allosteric effector, *e.g*., by shifting the equilibrium to the closed state to facilitate an efficient reaction. UDP-GlcNAc could act as such an allosteric effector. In some enzymes, the equilibrium between open and closed is so strongly shifted to the open state, that no reaction is observed without the allosteric UDP-GlcNAc (Samuel and Tanner, 2004; de Azevedo and Nascimento, 2019). The absence of the necessity of UDP-GlcNAc as an allosteric activator for UDP-ManNAc epimerization was first revealed by biochemical data for the *N. meningitidis* epimerase SacA and confirmed in the present study for the *P. alvei* epimerase MnaA. However, this seems to be an exception rather than a rule among UDP-GlcNAc-2-epimerases (Table 1). Interestingly, *Pa*MnaA shows a significantly higher product turnover and lower affinity for UDP-GlcNAc than the more strongly regulated enzymes (Velloso et al., 2008; Mann et al., 2016; Rattinam et al., 2022; Kawamura et al., 1978; Samuel and Tanner, 2004). Allosteric variants of *Pa*MnaA, Q42A, Q69A and H241A, significantly influence product turnover (Table 5), suggesting that these allosteric residues positively affect the rate of product formation and act less as traditional regulators.

Notably, among the tested UDP-GlcNAc-2 epimerases, only *Pa*MnaA is unaffected by tunicamycin (Figure 6). Despite of the importance of antibiotics in the design of novel strategies to counter significant pathogens, the molecular basis of inhibition of UDP-GlcNAc-2 epimerases by tunicamycin has not been elucidated at a structural level; no co-crystal structure with the inhibitor has been published, implicating that it is unknown at which site of the enzyme the antibiotic binds.

Tunicamycin, a natural product structurally related to UDP-GlcNAc, is a tight-binding competitive inhibitor targeting multiple UDP-GlcNAc binding enzymes (Vocadlo et al., 2022). The concentration of the antibiotic plays a critical role in its binding to targets. At higher concentrations, tunicamycin binds MraY, a UDP-*N*-acetylmuramoyl-pentapeptide: undecaprenyl-phosphate phospho-*N*-acetylmuramoyl-pentapeptide transferase involved in peptidoglycan biosynthesis (Ikeda et al., 1991); at lower concentrations, it selectively inhibits TarO, the first enzyme in WTA biosynthesis (Hancock et al., 1976; Richter et al., 2013), as well as *Sa*MnaA and Cap5P UDP-GlcNAc 2-epimerase, with tunicamycin binding demonstrated using saturation transfer difference nuclear magnetic resonance spectroscopy (Mann et al., 2016). The docking of tunicamycin to the allosteric site of *Sa*MnaA supports the binding of the antibiotic to the allosteric-site residues of the enzyme (Figure 7B). In contrast, for *Pa*MnaA, which showed no inhibition by tunicamycin at any tested concentration (Figure 6), different residues at the predicted allosteric site appeared exposed to the antibiotic in our docking approach (Figures 7A, 8A). Furthermore, docking of tunicamycin over the whole protein *Pa*MnaA (Supplementary Figure S12A) supports the lack of inhibition, with the simplest structural explanation being that the compound does not bind to *Pa*MnaA. Notably, the docking of tunicamycin to *Nm*SacA revealed an even different situation regarding the exposure of amino acid residues to tunicamycin (Supplementary Figure S11). Since no data about the sensitivity of *Nm*SacA towards tunicamycin exist in the literature, it remains to be investigated, if there is a possible connection between the reduced influence of allosteric activation and the lack of inhibition of epimerization by tunicamycin.

Notably, there are reports in the literature about inhibitors of bacterial UDP-GlcNAc 2-epimerases other than tunicamycin. For example, transition-state analogs (Stolz et al., 2004a; Stolz et al., 2004b), which strongly bind to the active site of the enzyme but exhibit only poor physiochemical properties due to their chemical nature. A promising inhibitor is the oxo-imidizolyl compound Epimerox, which targets the allosteric site of the UDP-2-GlcNAc epimerase in *B. anthracis* and demonstrated low-level resistance (Schuch et al., 2013).

## 5 Conclusion

The unliganded crystal structure of *Pa*MnaA reveals that the enzyme adopts an open conformation characterized by an accessible cleft between the N- and C-terminal domains. Despite the conservation of residues involved in binding the allosteric activator UDP-GlcNAc, *Pa*MnaA is not strictly regulated by the substrate, similar to *Nm*SacA. Unlike other UDP-GlcNAc 2-epimerases, the activity of *Pa*MnaA remains unaffected by tunicamycin.

Obtaining liganded co-crystal structures of *Pa*MnaA is an important future step in understanding the molecular recognition of substrate in UDP-GlcNAc 2-epimerases. Many aspects of this process remain uncertain. For example, it is unclear how the allosteric site discriminates between the epimers UDP-GlcNAc and UDP-ManNAc, while the catalytic site does not.

Expanding our understanding of these processes may contribute to providing a platform for the rational design of Gram-positive inhibitors, which in turn could selectively target essential cell wall biosynthetic enzymes, such as MnaA, involved in producing the exclusive targets that S-layer proteins recognize and bind, and would thus represent a new class of antimicrobial therapeutics.

## Data Availability

The X-ray diffraction data and structure presented in this study can be found online at the RCSB Protein Data Bank repository under entry 9CM8 (https://www.rcsb.org/structure/9CM8).
