# Supplementary material for: Insights into structure and activity of a UDP-GlcNAc 2-epimerase involved in secondary cell wall polymer biosynthesis in Paenibacillus alvei
Source: Front Mol Biosci. 2024 Sep 26;11:1470989. doi: 10.3389/fmolb.2024.1470989 (PMC11464976; doi:10.3389/fmolb.2024.1470989)
Supplement: Supplementary file 1 [file Image1.pdf]

Supplementary Material

Insights into structure and activity of a UDP-GlcNAc 2-epimerase involved in secondary cell wall polymer biosynthesis in *Paenibacillus alvei*

Cordula Stefanović, Max S. G. Legg, Nick Mateyko, Jakob Ender, Tea Kuvek, Chris Oostenbrink, Christina Schäffer, Stephen V. Evans, Fiona F. Hager-Mair

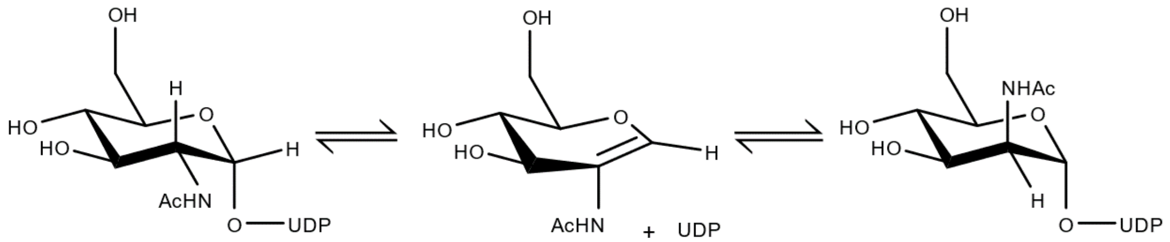

UDP-α-D-GlcNAc 2-acetoamidoglucal UDP-α-D-ManNAc

Supplementary FIGURE S1 Non-hydrolyzing UDP-GlcNAc 2-epimerases catalyze the reversible epimerization of UDP-α-D-GlcNAc at the C2 position yielding to UDP-α-D-ManNAc. This occurs *via* *anti*-elimination of UDP to a 2-acetoamidoglucal intermediate followed by *syn*-addition of UDP to yield the product UDP-ManNAc. This reaction is catalyzed by MnaA of *P. alvei* CCM 2051^{T} and involved in the biosynthesis of the bacterium's SCWP.

MSKVKVMTVF GVRPEAIKMA PLILELQKHP EHIESIVCVT AQHRQMLDQV LDVFNIKPDY
DLDIMQARQT LNDISVRVLQ GLEPVLQEAK PDIVLVHGDT LTTFLASYAA FMQQIKVGHV
EAGLRTWNKL SPFPEEMNRQ LTGVLADLHF APTDWSASNL RKENKQDASI FVTGNTVTDV
FQYTVRSDYK HEVLDWAQGK RLVLMTAHRR ESQGEPHRNI FRAVRRIADE FEDIAIVYPV
HPSPAVREPA HEMLGDHPRI KLIDPLDVVD LHNFYPHTHL ILTDSGGLQE EAPSFGIPVL
VLRETTERPE GIDAGTLELV GTDEEKVYAR AKALLSDETT YARMSKAANP YGDGKASQRI
VSAILHSFGV LEERPEPFHT KFTNLEHHHH HH

Supplementary FIGURE S2 Amino acid sequence of the MnaA construct used in this study. MnaA residues (1-384) are indicated in black font. The Leu-Gly linker and C-terminal His_6-tag derived from the pET22b(+) expression vector are indicated in grey.

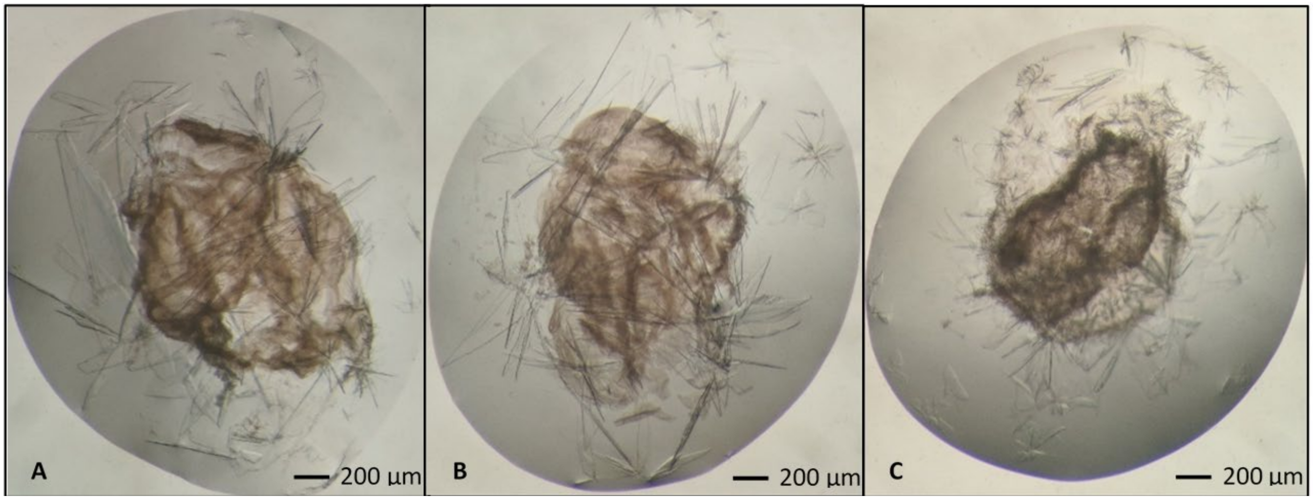

Supplementary FIGURE S3 MnaA crystal optimization with increasing PEG 4000 concentration. All conditions contained 0.2 M $CaCl_2$ and 0.1 M Tris/HCl, pH 8.5. The mother liquor-to-protein ratio was 1:1 (v/v). (A) 4% (w/v) PEG 4000, (B) 14.5% (w/v) PEG 4000, and (C) 15% (w/v) PEG 4000. The hanging drop method was used, and plates were stored at 18°C.

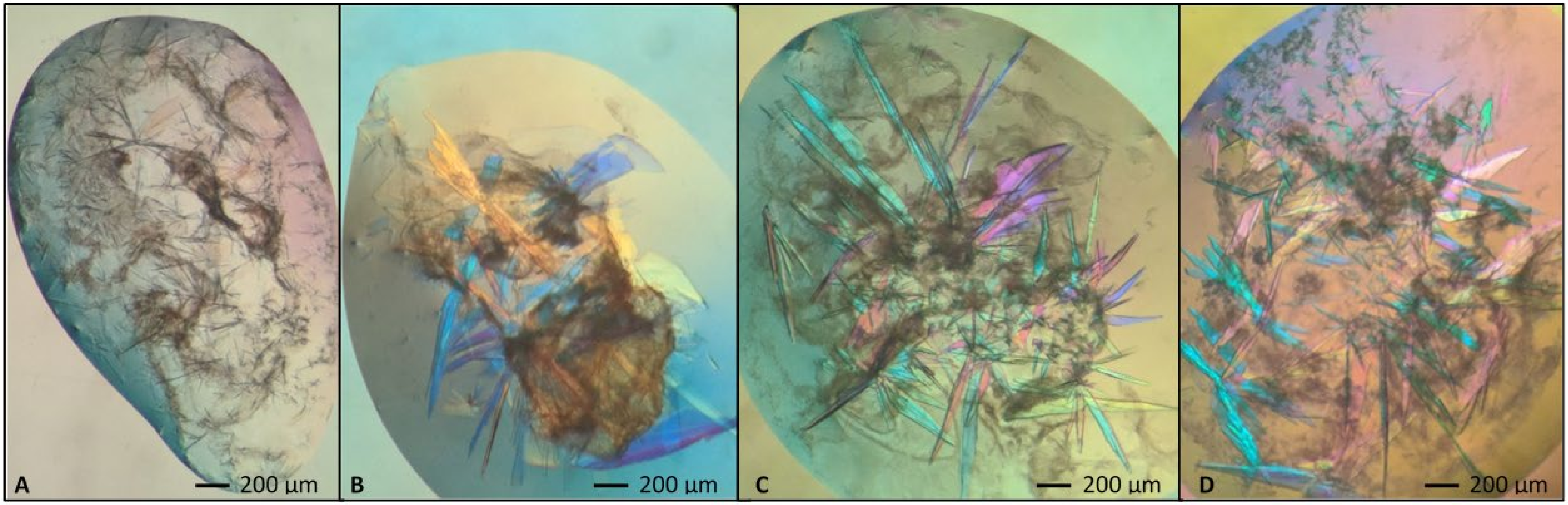

Supplementary FIGURE S4 MnaA crystal optimization with increasing drop size. All conditions contained 0.2 M $CaCl_2$, 0.1 M Tris/HCl, pH 8.5 and 15% (w/v) PEG 4000. The mother liquor-to-protein ratio was 1:1, with different volumes: (**A)** 0.5 µL, **(B)** 1 µL, (**C)** 1.5 µL, and (**D)** 2 µL. The hanging drop method was used, and plates were stored at 18°C.

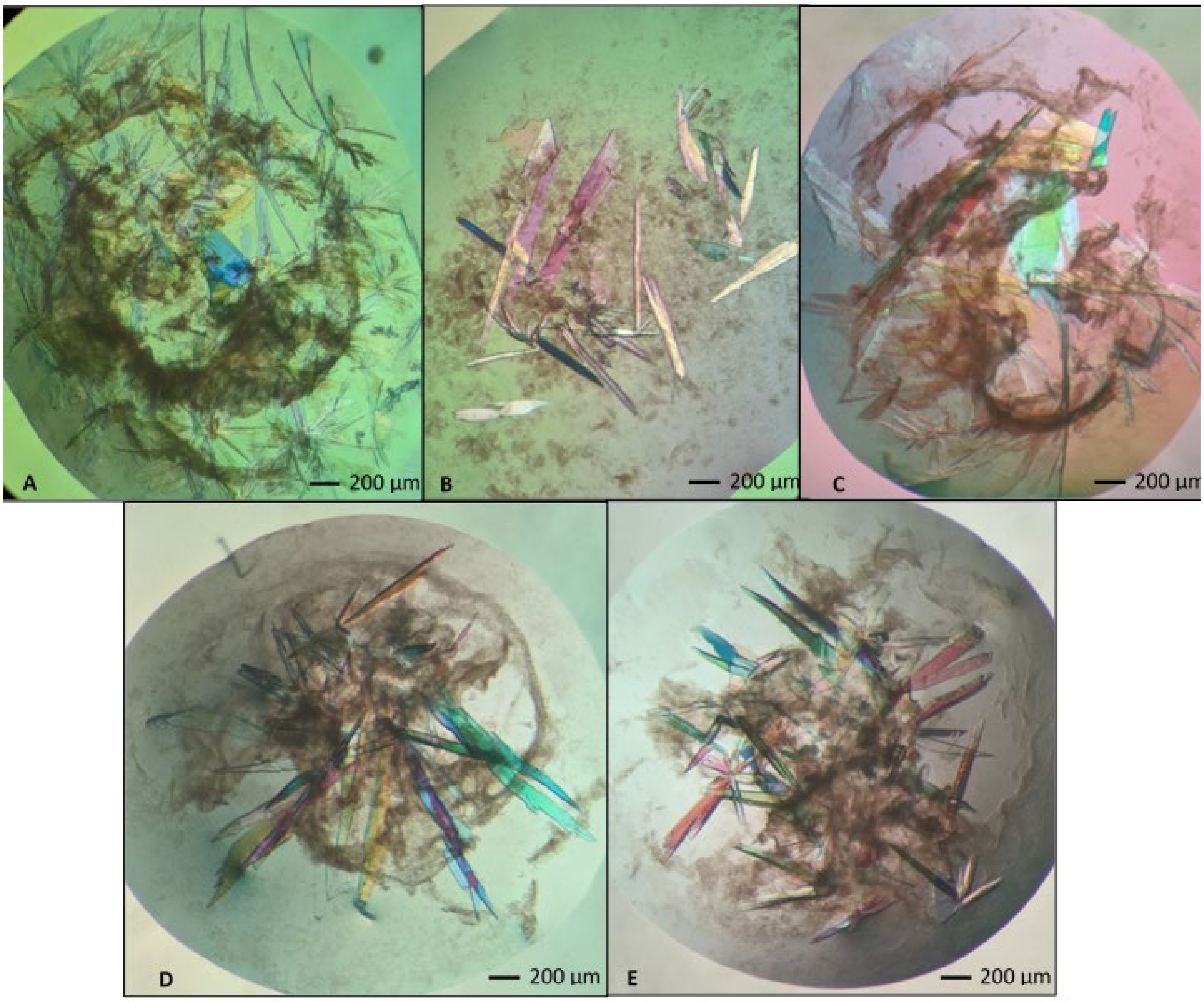

Supplementary FIGURE S5 MnaA crystal optimization with increasing pH. All conditions contained 0.2 M $CaCl_2$, 0.1 M Tris/HCl of varying pH, and 15% (w/v) PEG 4000. The mother liquor-to-protein ratio was 1:1 (1.5 µL). **(A)** pH 8.3, **(B)** pH 8.4, **(C)** pH 8.5, **(D)** pH 8.6, and **(E)** pH 8.7. The hanging drop method was used, and plates were stored at 18°C.

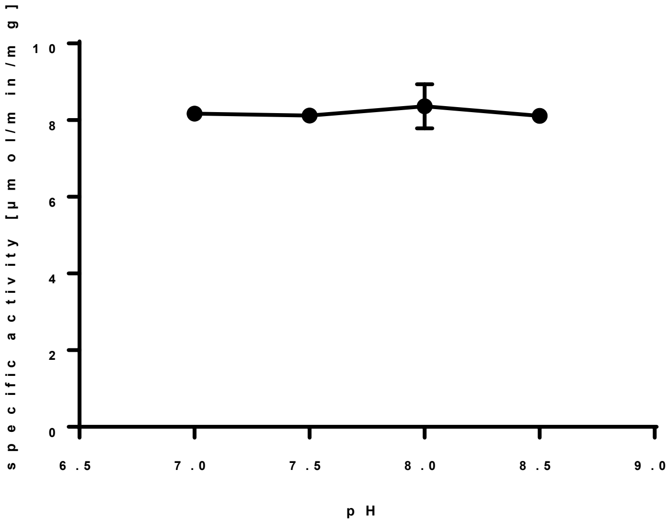

Supplementary FIGURE S6 Specific activity of *Pa*MnaA at a pH off 7, 7.5, 8 and 8.5. The analysis was done in duplicate measurements.

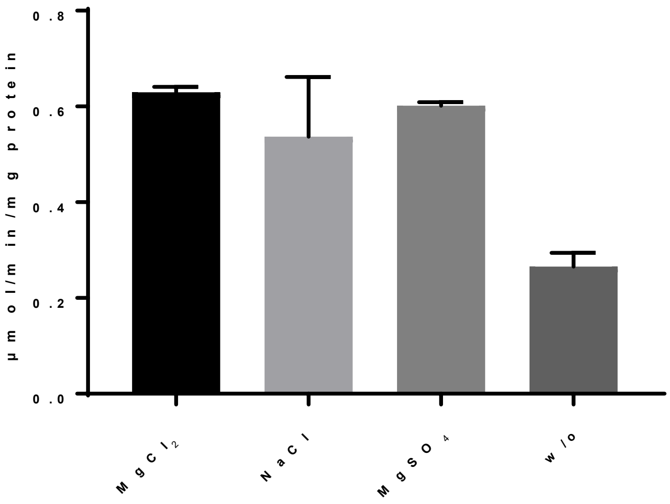

Supplementary FIGURE S7 Activity UDP-GlcNAc-epimerization by *Pa*MnaA (forward reaction) in the presence of $MgCl_2$, NaCl, and $MgSO_4$ 50 mM, each, or without salt addition (w/o). Measurements were performed in duplicate.

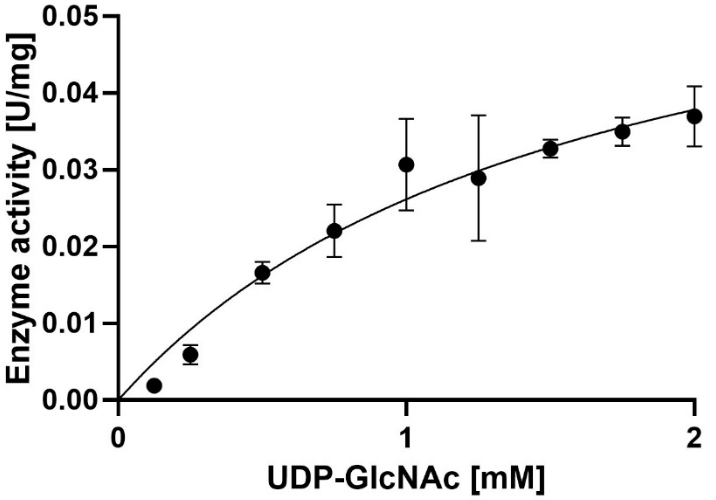

Supplementary FIGURE S8 Michaelis-Menten curve for the epimerization of UDP-GlcNAc to UDP-ManNAc (forward reaction) with the *Pa*MnaA variant Q42A (forward reaction, 840 ng).

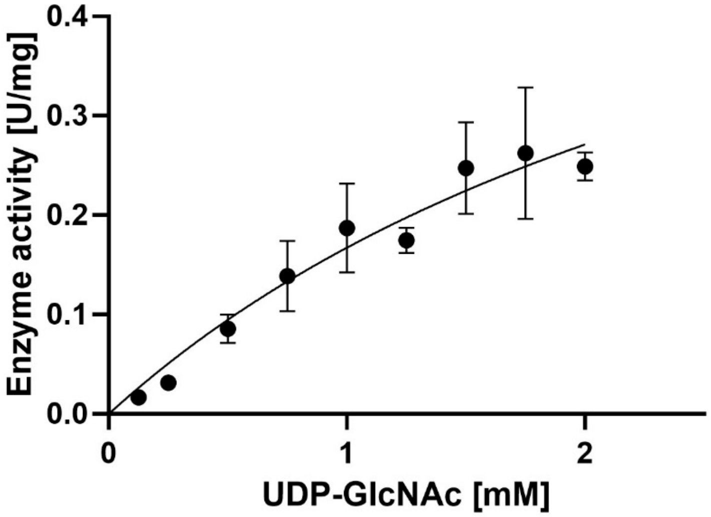

Supplementary FIGURE S9 Michaelis-Menten curve for the epimerization of UDP-GlcNAc to UDP-ManNAc (forward reaction) with the *Pa*MnaA variant H241A (forward reaction, 220 ng).

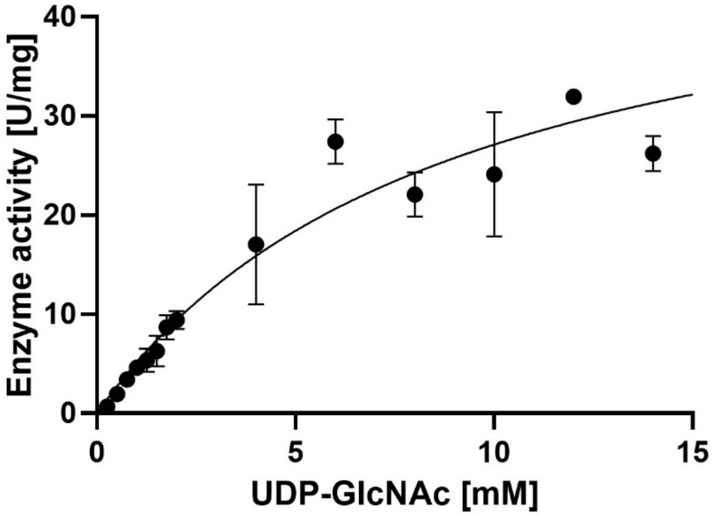

Supplementary FIGURE S10 Michaelis-Menten curve for the epimerization of UDP-GlcNAc to UDP-ManNAc (forward reaction) with the *Pa*MnaA variant Q69A (forward reaction, 52 ng).

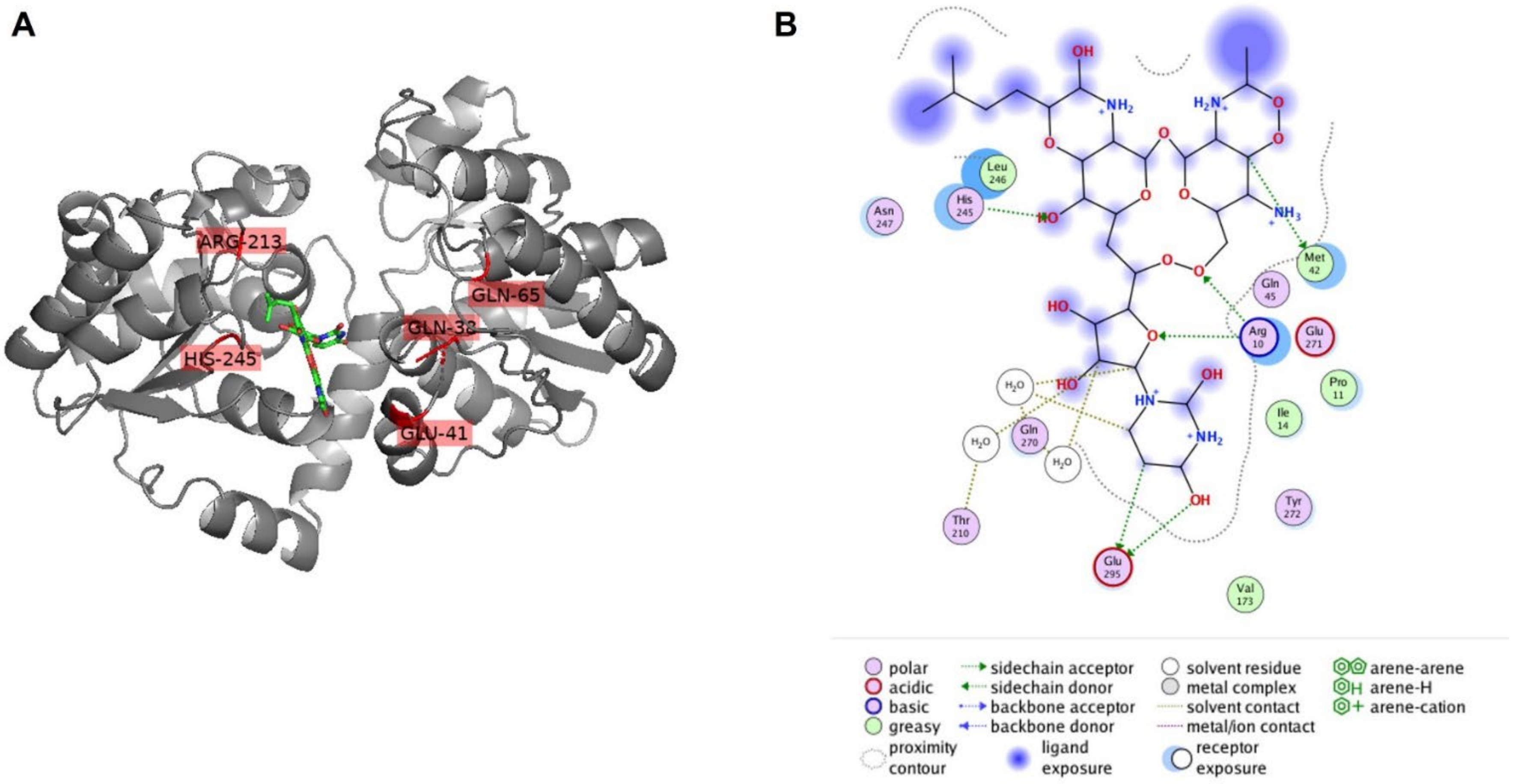

Supplementary FIGURE S11 **(A)** Docking of tunicamycin to the UDP-GlcNAc-2 epimerase of *N. meningitidis*. *Nm*SacA molecule A (6VLC) with conserved residues (Q39, H03, Q66, R206 and H238) is highlighted in red; residues were visualized by PyMoL (Open Source Version 2.4; https://github.com/schrodinger/pymol-open-source). **(B)** Ligand interaction diagrams for tunicamycin docked to predicted allosteric-site residues of *Nm*SacA (6VLC). Interacting residues of the enzymes are within a distance of 4.5 Å to the antibiotic. Interaction diagrams were calculated in MOE (Molecular Operating Environment, v2019).

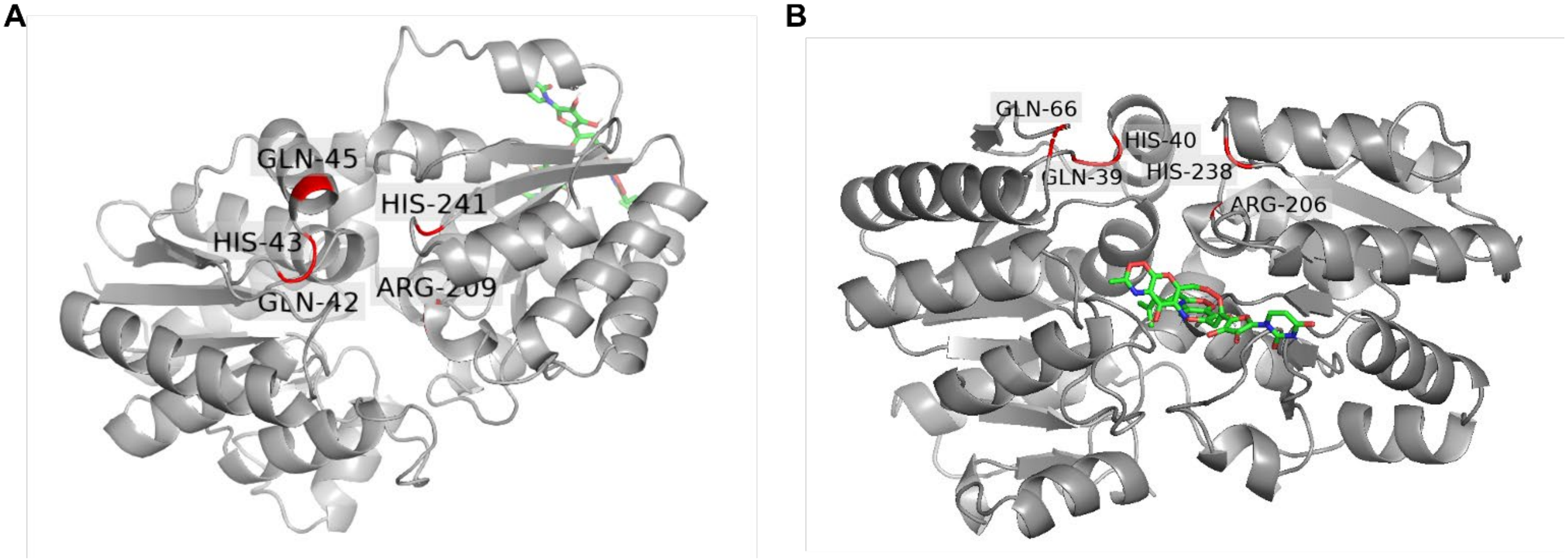

Supplementary FIGURE S12 Docking of tunicamycin (green) to a closed-conformation model of the UDP-GlcNAc-2 epimerase of *P. alvei* compared to *S. aureus* over the whole protein. **(A)** *Pa*MnaA molecule A modeled by Swiss Modell with docked tunicamycin into the allosteric site. Conserved residues (Q42, H43, Q45, Q69, R209 and H241) are highlighted in red; **(B)** *Sa*MnaA molecule A (5ENZ) with docked tunicamycin into the allosteric site with conserved residues (Q39, H03, Q66, R206 and H238) highlighted in red. Residues were visualized by PyMoL (Open Source Version 2.4; https://github.com/schrodinger/pymol-open-source).
